# Using Long-Term Follow-Up Data to Classify Genetic Variants in Newborn Screened Conditions

**DOI:** 10.3389/fgene.2022.859837

**Published:** 2022-05-26

**Authors:** Kevin Wilhelm, Mathew J. Edick, Susan A. Berry, Michael Hartnett, Amy Brower

**Affiliations:** ^1^ Newborn Screening Translational Research Network, American College of Medical Genetics and Genomics, Bethesda, MD, United States; ^2^ Graduate Program in Genetics and Genomics, Graduate School of Biological Sciences, Baylor College of Medicine, Houston, TX, United States; ^3^ Michigan Public Health Institute, Okemos, MI, United States; ^4^ Department of Pediatrics, Division of Genetics and Metabolism, University of Minnesota, Minneapolis, MN, United States

**Keywords:** newborn screening, longitudinal data, inborn errors of metabolism, newborn screening translational research network (NBSTRN), longitudinal pediatric data resource (LPDR), clinvar, variant classification, American college of medical genetics and genomics (ACMG)

## Abstract

With the rapid increase in publicly available sequencing data, healthcare professionals are tasked with understanding how genetic variation informs diagnosis and affects patient health outcomes. Understanding the impact of a genetic variant in disease could be used to predict susceptibility/protection and to help build a personalized medicine profile. In the United States, over 3.8 million newborns are screened for several rare genetic diseases each year, and the follow-up testing of screen-positive newborns often involves sequencing and the identification of variants. This presents the opportunity to use longitudinal health information from these newborns to inform the impact of variants identified in the course of diagnosis. To test this, we performed secondary analysis of a 10-year natural history study of individuals diagnosed with metabolic disorders included in newborn screening (NBS). We found 564 genetic variants with accompanying phenotypic data and identified that 161 of the 564 variants (29%) were not included in ClinVar. We were able to classify 139 of the 161 variants (86%) as pathogenic or likely pathogenic. This work demonstrates that secondary analysis of longitudinal data collected as part of NBS finds unreported genetic variants and the accompanying clinical information can inform the relationship between genotype and phenotype.

## 1 Introduction

From the development of Sanger Sequencing in 1977 (Sanger et al., 1977) to the advent of Next-Generation Sequencing (NGS) in 2005 (Shendure et al., 2005), the availability of low-cost genetic information has markedly expanded. As of 13 September 2021, the NCBI Reference Sequence Database (RefSeq) reported the submission of 40, 213, 945 transcript reads across 113,002 organisms (O’Leary et al., 2016). With the obstacles of high sequencing cost and intensive labor to generate data mostly overcome, genomics faces new hurdles: the interpretation and use of genetic variants to aid clinical decision-making (Krier et al., 2016). The importance of determining genotype-phenotype correlations to impact health outcomes has been reported in many publications (Trefz et al., 1993; Arnold et al., 2010; LD et al., 2016; Hsu et al., 2019) and current efforts to interpret genotype-phenotype correlations prefer to use population-specific biobanks, such as the All of Us Program (Denny et al., 2019) and the UK Biobank (Sudlow et al., 2015). The mining of these biobanks for variant and health information is a valuable resource for informing the relationship between genotype and phenotype, and improving the treatment, management, and health outcomes in individuals with a genetic disease.

To investigate another resource for determining the clinical relevance of variants, we conducted secondary analysis of a longitudinal data set of individuals identified with a rare genetic disease through newborn screening (NBS) for information about treatment and disease course. In the United States, NBS is a multi-component system of prenatal education, neonatal screening, clinical referral and diagnosis, and long-term medical management. A federal advisory committee recommends which conditions to screen, but the composition of screening panels is determined by state based NBS programs. The majority of screened conditions are inborn errors of metabolism (IEM), and 44 IEM disorders are currently included in the Recommended Uniform Screening Panel (RUSP) (Federal Advisory Committees, 2021). Variant and health information from a completed, 10-year natural history study of IEMs, called Inborn Errors of Metabolism Collaborative (IBEMC) (Berry et al., 2010; SA et al., 2016), was analyzed to find unpublished variants and review health information. The IBEMC dataset provides the potential for variant interpretation (Pena et al., 2016) using data from subjects that have had genetic testing for their condition and information about their clinical course collected over time.

## 2 Materials and Methods

### 2.1 Newborn Screening Translational Research Network (NBSTRN)

NBSTRN is a resource for investigators engaged in newborn screening related research led by the ACMG and is funded by a contract from the Eunice Kennedy Shriver National Institute of Child Health and Human Development (NICHD) and is a key component of the NICHD Hunter Kelly Newborn Screening Research Program (U.S. Code, 2021). The NBSTRN develops data tools and resources to facilitate both primary and secondary research efforts (Lloyd-Puryear et al., 2019) (https://nbstrn.org/). This effort utilized the Longitudinal Pediatric Data Resource (LPDR), one of the NBSTRN data tools housed in a Federal Information Security Modernization Act (FISMA) moderate environment, for the secondary analysis of the IBEMC data set (IBEMC MCAD Cohort; IBEMC PKU Cohort).

### 2.2 Inborn Errors of Metabolism Information System (IBEM-IS)

To discover unpublished genetic variants that may be implicated in the manifestation of IEMs, data from the Inborn Errors of Metabolism Information System (IBEM-IS) were examined. The IBEM-IS data were collected and managed in the IBEM-IS at Michigan Public Health Institute. The data set included phenotypic and genotype data on individuals with one of 42 NBS screened disorders. The original study was observational, resulting in only a subset of cases reported as having a genotype based on the following three factors as reported by the IBEMC: 1) the clinical relevance of genotyping as determined by the clinician, 2) the willingness of insurance providers to cover genotyping, and 3) the desire of patients to know his/her genotype (SA et al., 2016). The IBEM-IS collects information from subjects that could be used for secondary analysis and includes data categories such as demographic information, disease presentation, clinical diagnosis, treatments and interventions (Berry et al., 2010; SA et al., 2016). At the conclusion of the 10-years study, the IBEMC dataset was deidentified and transferred to the LPDR for secondary use by the research community. We accessed the IBEM-IS via the LPDR on 10 July 2018, and successfully analyzed data from 32 diseases and 1904 subjects.

### 2.3 Classification Guidelines

ClinVar, a repository of genetic variants and their correlation to medically important phenotypes (MJ et al., 2018), was used as the reference database for variants. Multiple publications have noted the importance of updating ClinVar with newly discovered variants and its importance in understanding the clinical implications of human variation (Harrison et al., 2016; Danos et al., 2018; Wain et al., 2018; Wei et al., 2018) Using ClinVar as a reference for published genotypes, each gene data set was exported from ClinVar for genes associated with diseases in the IBEM-IS from November 28–29, 2018, with the exception of Citrullinemia (CIT), extracted on 14 November 2018.

According to ClinVar (National Library of Medicine, 2019), submissions must assign standard terms for clinical significance as designated by ACMG/AMP (Richards et al., 2015) and this includes assignments for the consequence of the variant as Benign, Likely Benign, of Uncertain Significance, Likely Pathogenic, or Pathogenic. Although ClinVar establishes these terms as standard formats for reporting clinical significance, ClinVar does not calculate nor verify the assignment of these terms to submitted variants (Representation of clinical significance in ClinVar and other variation resources at NCBI). ClinVar designates the task of assigning a clinical significance term to the submitter, with exceptions for submissions from OMIM and early submissions before standard terms were required. In these instances, ClinVar calculated and verified the clinical significance of submitted variants.

We used the American College of Medical Genetics and Genomics (ACMG) and the Association for Molecular Pathology (AMP) variant interpretation guidelines (Richards et al., 2015), to build evidence for accurate variant classification and used the IBEM-IS data points shown in Table 1. The ACMG/AMP publication provided a method for ascertaining the strength of evidence for determining a variant’s correlation with a disease phenotype. Points of evidence include population data, computational and predictive data, functional data, segregation data, *de novo* data, allelic data, other databases, and other data. Varying types of data and observations correlate to either pathogenic or benign criteria, which are incorporated into the final determination of significance. The classification criteria used in this analysis can be seen in Table 2. PS3 (functional assay) and PP4 (well-characterized phenotype) criteria were assigned to 161 unpublished variants found in the IBEM-IS dataset, due to each patient in the data set having a confirmatory diagnostic test and well-known disorder. Unmapped variants were not assigned any criteria. Unpublished variants are described as variants that have not been submitted to ClinVar and unmapped variants are variants that did not map to any transcripts listed in the RefSeq database. Because all subjects enrolled in IBEMC were diagnosed using functional blood metabolite or enzyme assays through their newborn screen and confirmatory diagnostic testing, the variants for these subjects were classified as PS3. All diseases in the IBEMC study have been well-characterized and display a specific early-onset phenotype, deserving the attribution of PP4. All other criteria were determined based on the clinical information available for each variant. Mutalyzer (Lefter et al., 2021), a web-based tool for mapping variants to reference sequences, was used to validate the unpublished variants found in the IBEM-IS (Supplementary Table S1). ClinVitae (Invitae | Clinvitae, 2019) was used as a secondary source of published variants. ClinVitae is a database reporting variants from Clinvar, Emory Genetics Laboratory Variant Classification Catalog, Invitae, ARUP Mutation Databases, Kathleen Cunningham Foundation Consortium, and Carver Mutation Database. FATHMM (Shihab et al., 2013) and SNPS&GO (Calabrese et al., 2009; Capriotti and Altman, 2011) web-based computational prediction tools were used to predict the functional effects of variants reported. FATHMM is a web-based evolutionary conservation prediction tool that is used to predict the functional consequence of both coding and non-coding variants. SNPS&GO is a web-based protein structure/function prediction tool that assesses the functional impact of coding variations.

**TABLE 1:** Variant classification criteria and supporting data Source(s). The ACMG/AMP Evidence-Based Criteria (Richards et al., 2015) was used to determine supporting data sources. No supporting data was generated for the “Population Data” criteria defined by the ACMG/AMP guidelines. Supporting data for other evidence-based criteria were found using computational tools (Calabrese et al., 2009; Capriotti and Altman, 2011; Shihab et al., 2013), within in the long-term follow-up dataset (Segregation Data, De novo Data), reported by other databases (Invitae | Clinvitae, 2019), or assumed from the nature of newborn screening/the disease (Functional Data, Other Data).

ACMG/AMP evidence-based criteria	Supporting data source
Population Data	No population data was generated
Computational and Predictive Data	FATHMM(Shihab et al., 2013), SNPS&GO (Calabrese et al., 2009; Capriotti and Altman, 2011)
Functional Data	All cases confirmed by newborn screen and supplemental testing
Segregation Data	Family history
De novo Data	Family history
Allelic Data	For autosomal recessive disorders, it is assumed that reported variants were reported *in trans*
Other Database	ClinVitae (Invitae | Clinvitae, 2019)
Other Data	Analyzed disorders have been established as genetically based, supporting a distinctive phenotype for gene

**TABLE 2:** Number of variants assigned a pathogenicity criterion. The ACMG/AMP guidelines have various clinical significance criteria, that when combined, result in a clinical significance classification. ACMG/AMP scoring criteria are show on the left, with the number of variants assigned that criteria shown on the right. Percentages calculated from the total number of unpublished variants (n = 161).

ACMG/AMP evidence found in LPDR	Number of variants (n = 161)
PVS1 (Null Variants)	43 (26.7%)
PS3 (Functional Studies)	150 (93.1%)
PM3 (Cis/trans confirmation)	66 (41.0%)
PM5 (Novel missense at same position as published pathogenic variant)	13 (8.1%)
PM6 (De novo)	2 (1.2%)
PP1 (Segregation Analysis)	7 (4.3%)
PP3 (Computational *in silico* data)	77 (47.8%)
PP4 (Phenotype to support variant)	150 (93.1%)
PP5 (Found in reputable database)	23 (14.3%)
BP4 (Computational *in silico* data)	1 (0.6%)
BP7 (Synonymous variants)	4 (2.5%)

### 2.4 Pipeline Structure

To analyze the IBEM-IS data within the LPDR, a Python-based (v2.7.16) (Python, 2019) script was used to extract patient information and compare variants to ClinVar. Python is a high-level, object-oriented programming language allowing users to interact with dynamic data and interface with open-source libraries. Much of the script utilized data frames and analysis tools provided by Pandas library. Pandas is an open-source Python package used to analyze structured data and is considered a powerful data manipulation and analysis tools (pandas, 2019). The script references the IBEM-IS data set and ClinVar gene extracts through saved comma-separated values (CSV) files. The pipeline was built around essential processes, that were needed to analyze the data thoroughly and are expanded upon in the following sections.

#### 2.4.1 Review Case Level Data

The IBEM-IS has over 8,228 subjects reporting longitudinal data distributed across 7,300 data fields. To facilitate data set analysis, the entire IBEM-IS dataset was divided into disorder category tables (amino acid disorders, fatty acid oxidation disorders, etc.) then subsequently further divided into disease-specific tables. In addition to making the data set more manageable, this process helps to confirm that a patient’s diagnosis was submitted correctly. Once the data was sorted, the total number of subjects with the disease was calculated and each patient’s record was checked for the submission of a variant. In IBEM-IS, variants were reported in one of two formats: 1) the selection of published genotypes and 2) a custom text submission. Variants at this stage were also checked for nonvalid variant submissions, such as “none” or “negative”, to streamline comparison to ClinVar extracts. If a variant was found in the patient’s record, it was saved and used for comparison.

#### 2.4.2 Convert ClinVar Variants

ClinVar reports variants using the Human Genome Variation Society (HGVS) format, which describes the genetic variant (i.e. c.549A > C) and the resulting protein variant (i.e., p. Phe256Leu)^27^. ClinVar also requires that the variant be submitted containing the reference sequence accession code to which the variant was mapped. There was not a uniform variant reporting format in the IBEM-IS data and most submissions consisted of only a genetic or a protein variant, not including both elements of the HGVS format. When included as protein variants, most variants were reported using single-letter amino acid codes and position in the protein, i.e. F256L. The HGVS segment in the ClinVar variant was converted to the single-letter amino acid code format to reconcile the two protein reporting formats during analysis.

#### 2.4.3 Compare Genotypes to ClinVar Database and Deduplicate

Variants found in the IBEM-IS were compared to published ClinVar variants. If the IBEM-IS variant matched a ClinVar variant, the variant was appended to the disease-specific published list. If the IBEM-IS variant did not match a ClinVar variant, the variant was appended to the disease-specific unpublished list. The records containing variants not found in ClinVar were manually re-checked and used for the next step in the pipeline.

#### 2.4.4 Extract Clinical Data

When a variant was not found in ClinVar, the patient’s record was searched for clinical data. Clinical data of interest were NBS result, family history, treatment, medical management, and allelic (*cis*/*trans* testing) data to aid in determination of recessive phenotypes. These clinical data points were selected according to the ACMG/AMP guidelines (Richards et al., 2015). If clinical data was discovered in the patient’s record, it was extracted and saved.

#### 2.4.5 Output Check and Variant Classification

To archive all results obtained from the pipeline, an output text file (.txt) was saved with information for each disease. The output text file contains the clinical data associated with each variant, the locally compiled published and unpublished list of variants, and the total number of subjects found in the disease-specific table. After the output text file is exported, a manual check of variants is needed to ensure variant comparison accuracy. After the output verification, the information was compiled for pathogenicity classification using the ACMG/AMP guidelines. Classified variants will be submitted to the ClinVar repository.

### 2.5 Time-Stamped Analysis

To perform a time-stamped analysis, ClinVar was searched on 1 October 2021 for the 33 genes in which the 150 variants were classified. ClinVar records were searched by gene name and all variants associated with the gene were downloaded. The 150 classified variants in this study were checked for inclusion in the updated ClinVar search. Variants that were found were analyzed for classification accuracy by comparing the ClinVar classification to the classification given in this study.

## 3 Results

### 3.1 LPDR Data Summary

2,124 subjects were enrolled in the IBEM-IS when the data was transferred to the LPDR for secondary use. Of these enrolled, 1904 subjects had a diagnosis of one of the 32 diseases that were successfully analyzed to determine if genetic variants had been reported. Ten diseases were not analyzed due to either no genotype or unpublished variants reported for a patient. Genotyping was performed on 982 (51.6%) out of 1904 subjects with a diagnosis of one of 32 analyzed diseases. Of the analyzed diseases, 10 (31.3%) were categorized as amino acid disorders, 8 (25%) were fatty acid oxidation disorders, 11 (34.4%) were organic acid disorders, and 3 (9.4%) were categorized as other disorders. Table 2 lists the number of subjects for each condition and the categorization of variants in ClinVar. These data show that data collected by observational studies and maintained by the NBSTRN contain diverse disease data.

### 3.2 Classification of 150 Variants With Supporting Clinical Information

Among the 982 subjects where a genetic variant was recorded in the LPDR, 564 individual variants were identified. Of those variants, 403 (71.5%) were present in ClinVar and 161 (28.5%) variants were not found in the ClinVar database. The 161 unpublished variants were reported in 29 diseases, shown in Supplementary Table S2. The clinical data from subjects with these 161 variants was used to build evidence for variant-disease correlation. The breakdown of the ACMG/AMP scoring criteria assigned to unpublished variants is shown in Table 2. While mapping variants to reference sequences, 11 variants were discovered that were reported with an incorrect reference amino acid at the submitted protein residue position. These incorrect submissions were confirmed with FATHMM (Shihab et al., 2013) and SNPS&GO (Calabrese et al., 2009) (Supplementary Table S3, S4). These 11 variants were not further analyzed nor assigned a classification. The remaining 150 variants (93.1%) mapped to reference sequences were attributed PS3 and PP4 pathogenicity criteria due to the nature of the disease dataset being studied (Supplementary Table S5). Ninety-one variants were classified as Likely Pathogenic and were assigned using the “Likely Pathogenic 2” (one strong and one to two moderate) and “Likely Pathogenic 3” (one strong and more than two supporting) combination criteria. 41 variants were classified according to “Likely Pathogenic 2” and 50 were classified according to “Likely Pathogenic 3”. Moderate and supporting classification criteria were obtained from computational prediction (PM5 and PP3), discovery in other databases (PP5), segregation (PP1), *de novo* (PM6), and allelic (PM3) data. The distribution of variants assigned these criteria can also be found in Table 3. During analysis, 11 variants discovered did not have enough clinical information to assign a classification. These 11 variants were attributed with PS3 and PP4 classification criteria but did not have additional information necessary to determine a classification, thus, they remain as Variants of Uncertain Significance (VUS). These data show that the LPDR contains undescribed variants and the clinical data needed to classify them.

**TABLE 3:** Classification of the 161 unpublished variants according to ACMG/AMP guidelines. By combining the criteria shown in Table 3, variants were assigned a clinical significance. The classification definitions are: 1) Pathogenic, a variant that is “actionable” and may affect clinical decision making regarding management, treatment, or surveillance, 2) Likely Pathogenic, meaning “greater than 90% certainty of a variant … being disease-causing” (Richards et al., 2015), 3) Variant of Unknown Significance (VUS), meaning the data was either conflicting or did not report information that fulfilled the ACMG/AMP criteria, and 4) Unmapped variants, referring to variants in the data set that reported incorrect reference amino acids.

ACMG/AMP classification	Number of variants (n = 161)
Pathogenic (Criteria 1a)	44 (27.3%)
Pathogenic (Criteria 3b)	4 (2.5%)
Likely Pathogenic (Criteria 2)	41 (25.5%)
Likely Pathogenic (Criteria 3)	50 (31.1%)
Variants of Unknown Significance (VUS)	11 (6.8%)
Unmapped Variants	11 (6.8%)

Forty-eight of the 161 variants were found to have evidence supporting classification as Pathogenic. A total of 44 predicted null variants were discovered across 20 diseases, which were attributed with PVS1 pathogenicity criteria. PVS1 and PS3 attributed variants satisfied the “Pathogenic 1a” combination requirements for classifying the variant as Pathogenic. Four variants were classified as Pathogenic according to combination criteria for “Pathogenic 3b”, using two moderate (PM1-6) classification criteria and two supporting (PP1-PP5) criteria. These data show that the LPDR contains substantial numbers of pathogenic variants that have remained undescribed.

### 3.3 Time-Stamp Analysis Demonstrates the Continual Expansion of ClinVar

To determine whether our novel variants had been submitted to ClinVar since the original analysis, we performed an updated search of ClinVar (Methods) for variants in the 33 genes from our analysis. The updated search returned an additional 7,469 variants, resulting in a total of 14,556 variants (original plus updated). Of the 150 novel variants we classified in the original analysis, eight had since been submitted to ClinVar (Hypergeometric test; p = 1.61e-05). We compared the pathogenicity classification in ClinVar for the eight variants (Table 4). Four of the eight variants (*GCDH*:c.776C > T (p.Ser259Leu), *GCDH*:c.880C > T (p.Arg294Trp), *GALT*:c.601C > T (p.Arg201Cys), *ASL*:c.1366C > T (p.Arg456Trp)) were classified as Pathogenic or Likely Pathogenic in ClinVar and are additionally supported by the classification in this study. The remaining four of eight variants are classified as Uncertain Significance or Conflicting Interpretations of Pathogenicity in ClinVar. The time-stamp analysis demonstrates that ClinVar is a continually changing resource of genotype-phenotype characterizations and that data collections like the IBEM-IS contribute to this ongoing effort.

**TABLE 4:** Classifications of eight variants identified in time-stamp analysis. Eight variants classified in this study were submitted to ClinVar since the original search for submissions. The classifications assigned to the eight variants in ClinVar, as well as the review status, and in this study are shown. One star and two-star review statuses correspond to variants having criteria provided by a single submitter and criteria provided by multiple submitters without conflicting interpretations, respectively.

Variant	ClinVar classification	Study classification
NM_000159.4 (GCDH):c.776C > T (p.Ser259Leu)	Likely Pathogenic (Review Status: 1 star)	Likely Pathogenic
NM_000159.4 (GCDH):c.880C > T (p.Arg294Trp)	Pathogenic (Review Status: 1 star)	Likely Pathogenic
NM_000155.4 (GALT):c.601C > T (p.Arg201Cys)	Pathogenic (Review Status: 2 star)	Likely Pathogenic
NM_004453.4 (ETFDH):c.731T > C (p.Phe244Ser)	Uncertain Significance (Review Status: 1 star)	Likely Pathogenic
NM_000016.6 (ACADM):c.92G > A (p.Arg31His)	Uncertain Significance (Review Status: 2 star)	Uncertain Significance
NM_000018.4 (ACADVL):c.1019G > A (p.Gly340Glu)	Uncertain Significance (Review Status: 1 star)	Likely Pathogenic
NM_000018.4 (ACADVL):c.1838G > A (p.Arg613Gln)	Conflicting Interpretations of Pathogenicity (Review Status: 1 star)	Likely Pathogenic
NM_000048.4 (ASL):c.1366C > T (p.Arg456Trp)	Pathogenic (Review Status: 1 star)	Likely Pathogenic

## 4 Discussion

This study is the first to use secondary analysis of health information from a NBS longitudinal dataset housed in the LPDR to classify variants. In addition to collecting variant data used in the diagnosis of individuals, longitudinal databases also capture follow-up visits describing the treatment plan and additional clinical testing data. By analyzing these databases, we have the opportunity to expand our knowledge of genotype-phenotype correlations, determine the clinical relevance of variants, and reduce the number of VUSs complicating interpretation of variants in reference variant databases.

This work demonstrates that longitudinal data contained in resources like the NBSTRN LPDR should be considered of high value to the research and clinical communities. The LPDR offers a unique ability to access both NBS and clinical data of subjects with a confirmed diagnosis. The LPDR also offers another unique advantage to understanding genotype-phenotype correlations: subjects are followed from the neonatal period over an extended period with clinical data medical management over the lifespan of diagnosed individuals. This method of continuous data capture can be used to determine if patient genotypes are relevant to disease outcomes or could help direct clinical care based on past findings. The LPDR should, therefore, be useful in translating genetic variant findings into clinical action. While our effort focused on the secondary analysis of IEMs, the NBS community is beginning to accelerate efforts to capture long-term follow-up (LTFU) data on all NBS conditions. Methods and approaches like the one described here, can be applied to these new efforts to enhance broad understanding of clinical relevance of variant data captured in newborns and further inform public policy regarding the utility of genome sequencing in newborn screening.

Of note, the IBEM-IS did not mandate the use of HGVS variant in data capture and did not recommend any standardization of formatting. The lack of uniformity between variant submissions was a difficult task to overcome in this analysis. As more projects are completed and transferred to the NBSTRN for secondary research, the issue of non-interoperable variant submissions will worsen unless uniform requirements for data entry are promoted. As such, it is recommended that data tools like the LPDR work to educate researchers about standardized formats, such as the HGVS. Using a standardized format will allow researchers to spend less time cleaning data and help ensure the integrity of data within. As the amount of genetic variant data available continues to grow, researchers and clinicians will need data tools like the LPDR to determine the best care for individuals with a variant, offering detailed phenotypic correlations and presenting a valuable opportunity for corroboration of the clinical relevance of each genotype.

## Data Availability

The IBEMC dataset can be accessed through the Longitudinal Pediatric Data Resource (LPDR) hosted by the Newborn Screening Translational Research Network (NBSTRN) (NBSTRN, 2021a; NBSTRN, 2021b). The original contributions presented in the study are included in the article/Supplementary Materials, further inquiries can be directed to the corresponding author.

## Supplementary Material

Supplementary Table S1 Results of Sequence Variant Nomenclature according to the Human Genome Variation Society from Mutalyzer (Wildeman et al., 2008).

Supplementary Table S2 For each disorder, the number of subjects reporting a genotype are shown. Unique variants not found in ClinVar were assigned clinical significance and shown in the number of variants classified as Pathogenic, Likely Pathogenic, or remain as Variants of Uncertain Significance. Variants were assigned a clinical significance using data shown in Table 1.

Supplementary Table S3 Variant pathogenicity predictions provided by FATHMM (Shihab et al., 2013).

Supplementary Table S4 Variant pathogenicity predictions provided by SNPS&GO (Calabrese et al., 2009; Capriotti and Altman, 2011).

Supplementary Table S5 Detailed scoring assessment for each unpublished variant identified from the IBEMC.
